# Supplementary material for: The Effects of Heatwaves on Human Morbidity in Primary Care Settings: A Case-Crossover Study
Source: Int J Environ Res Public Health. 2022 Jan 12;19(2):832. doi: 10.3390/ijerph19020832 (PMC8775418; doi:10.3390/ijerph19020832)
Supplement: Supplementary file 1 [file ijerph-19-00832-s001.zip › ijerph-1474583-supplementary/Table S1_morbidities_summary statitics.pdf]

Average Number of Events Per Day in Heat and Non-Heatwave Days

ICPCC	HW		non-HW	
	X̄	IQR	X̄	IQR
	General and heat-related			
A04	3	2	4	3.75
A05	1	0	1	0
A06	1	1	1	1
A88	3	3	1	1
T11	1	0	1	0
K88	1	0	1	1
	Cardiovascular			
K74	1	0.25	1	0.75
K75	1	1	1	0.75
K76	1	1	1	0
K78	1	1	1	1
K79	1	0	1	0
K80	1	0	1	1
K89	1	1	1	0
K90	1	1	1	1
	Respiratory			
R02	1	1	1	1
URTI	38	43	50	59
LRTI	8	9	12	9
R80	4	3	5	4
R95	2	1	1	1
R96	3	4	4	4

Notes. X̄= median, IQR= interquartile range, ICPCC= International Classification of Primary Care code, HW= heatwave. For the meaning of the codes, refer to Table 1.
